# Seaweed cellulose scaffolds derived from green macroalgae for tissue engineering

**DOI:** 10.1038/s41598-021-90903-2

**Published:** 2021-06-04

**Authors:** Nurit Bar-Shai, Orna Sharabani-Yosef, Meiron Zollmann, Ayelet Lesman, Alexander Golberg

**Affiliations:** 1 Porter School of Environment and Earth Sciences, Tel Aviv University, Tel Aviv, Israel; 2 School of Biomedical Engineering, Tel Aviv University, Tel Aviv, Israel; 3 School of Mechanical Engineering, Tel Aviv University, Tel Aviv, Israel; 4 The Center for the Physics and Chemistry of Living Systems, Tel Aviv University, Tel Aviv, Israel

**Keywords:** Biomaterials, Tissue engineering

## Abstract

Extracellular matrix (ECM) provides structural support for cell growth, attachments and proliferation, which greatly impact cell fate. Marine macroalgae species *Ulva* sp. and *Cladophora* sp. were selected for their structural variations, porous and fibrous respectively, and evaluated as alternative ECM candidates. Decellularization–recellularization approach was used to fabricate seaweed cellulose-based scaffolds for in-vitro mammalian cell growth. Both scaffolds were confirmed nontoxic to fibroblasts, indicated by high viability for up to 40 days in culture. Each seaweed cellulose structure demonstrated distinct impact on cell behavior and proliferation rates. The *Cladophora* sp. scaffold promoted elongated cells spreading along its fibers’ axis, and a gradual linear cell growth, while the *Ulva* sp. porous surface, facilitated rapid cell growth in all directions, reaching saturation at week 3. As such, seaweed-cellulose is an environmentally, biocompatible novel biomaterial, with structural variations that hold a great potential for diverse biomedical applications, while promoting aquaculture and ecological agenda.

## Introduction

In native tissues, the Extracellular Matrix (ECM) is an essential platform that fulfills several functions, including providing structural support for cell growth, impact on cell behavior, and stimulating tissue regeneration^1^. Many of the challenges we face today are concerned with designing cost effective and safe alternatives to the technologies and materials currently in use to create microenvironments that would mimic the biochemical and physiological structures of natural environments within the human body^1^. Multiple fabrication strategies and material sources have been investigated as promising biomaterials with novel properties^2^. Among natural materials that serve as scaffolds for tissue engineering, cellulose-based matrices are relatively new to this research field, and are currently investigated to facilitate mammalian cell culture in vitro and in vivo^3^.

Cellulose is the most abundant polymer in nature and is a key structural element of the cell wall of plants, which gives the cell its mechanical strength and rigidity. In cotton, for example, it accounts for about 90% of the plant cell wall content. Together with lignin and hemicellulose, it supports plant’s vertical growth. It is a stable polymer that consists of tightly packaged glucose monomers, which provides it with a highly organized structure, difficult to break apart and unfavorable to biodegradation in the absence of cellulolytic enzymes^4,5^. These characteristics give cellulose unique biophysical and biomechanical properties, which are stable over a long time. As such, it can preserve its shape with minimal deformation, and function as a permanent construct or as a structural support, that could ideally be used as a template to guide the restructuring of cells and newly formed tissue for various applications, such as skin and wound dressings, bone tissue, blood vessels, neural, muscle, tendons, cartilage, vertebrae disks, urinary tracts, and larynx tissues, to name a few^3^. Moreover, cellulose hydrophilicity and fluid uptake could provide a moist construct for wound healing environment^6^**,** while promoting interaction with negatively charged cell surface, and thus advancing cell adhesion and proliferation^3^. Cellulose sources in tissue engineering range from natural polymers derived from plants^7–9^ and bacterial nanocellulose (BNC)^10^, to synthetic modified polymers^11^. These allow the diverse and versatile range of cellulose mechanical, physical, and structural properties such as morphologies, as a stand-alone or as a composite reinforcement material^3^*.* However, in order to fully fabricate cellulose-based biomimetic tissues that require specific cell–matrix interactions, further investigations of cellulose structural properties that could mimic native tissues are studied. For instance, cellulose derived from apple hypanthium was studied for adipose tissue engineering, carrot for bone tissue engineering, celery for tendons^9^ and BNC for burns and chronic wounds treatments^10^ or in vivo implantations^12^.

Alternatively, cellulose derived from macroalgae has not been fabricated as a standalone scaffold for tissue engineering. Macroalgae, known as seaweed, have a biostable structural and biochemical advantages, compared to bacterial and terrestrial plants cellulose, including high degree of cellulose crystallinity^4^ that results in higher inertness and makes seaweed cellulose less susceptible to chemical and thermal treatments^13^. Similar to plants, green macroalgae’s matrix consist of highly robust skeleton structure that can be utilized for cell growth. Their chemical composition is rich with insoluble polysaccharides, that provides for the preservation of structural and mechanical rigidity, crystallinity and tensile strength^13,14^. Thus, their structural and biochemical variations could potentially be considered for biomedical applications that do not require biodegradability, while maintaining intact shape and form. However, unlike bacterial based-cellulose, that require strong bases treatment for the removal of microbial cells^10^, and unlike terrestrial plants that require vertical growth^4^, seaweed lignin-free cell-wall makes macroalgae decellularization easier and cheaper to produce. Furthermore, it grants its matrix structurally flexible yet resilient tissue, which could potentially be explored for its ECM-cell interactions^15^and long term sustainability^4^.

Macroalgae have high growth rates, they are abundant and could be harvested easily all year with no need for fertilizers^16^, which makes its mass production more affordable and macroalgae a reliable low-cost resource. Additionally, macroalgae show environmental advantages. They do not compete with food supply, land for agriculture and forestry, or freshwater supply^17^. They help to mitigate global warming and climate change by utilizing doses of CO_2_. Common macroalgae derivatives are renewable and sustainable resources for food, fuel and chemicals applications^17^. Furthermore, among seaweeds, red and brown algae species are largely used for their carrageenans, alginates and agaroses in tissue engineering, wound healing and drug delivery^18^, and play a major role in biological and biomedical products^19^. Green macroalgae derived sulfated polysaccharides (SPs), such as ulvans, too, have been proposed for tissue engineering^20,21^. However, marine natural source of cellulose from green macroalgae have been overlooked for biomedical applications^3^.

Cellulose-based ECM is a relatively new field of research, more so macroalgae-based cellulose^22,23^, and little is known about its compatibility as an alternative matrix for cell culture. In the present study, we aim to understand the behavior and growth characteristic of fibroblasts cultured onto seaweed natural cellulose-based matrices. More specifically, we characterized and evaluated two marine green macroalgae species, *Ulva* sp. and *Cladophora* sp. We studied the natural structures of seaweed cellulose and compared between their distinct matrices: the first, a porous, comb-like structure^24^ and the second a fibrous, thread-like structure^13^. Finally, we examined the impact of each structure on cell fate, morphology and proliferation rates. As such, both seaweed structures could provide with a credible platform that supports cell growth and thus applied to a wide range of biomedical applications. From highly specialized membrane and carrier materials, to optimal biocompatible scaffolds, for wound healing, wound dressing and tissue engineering, that would on the one hand, require elasticity and strength, and on the other hand obtain intact permanent shape and form or long-term structural support, while promoting aquaculture and zero-waste agenda.

## Results

### Seaweed decellularization

**Figure 1:**
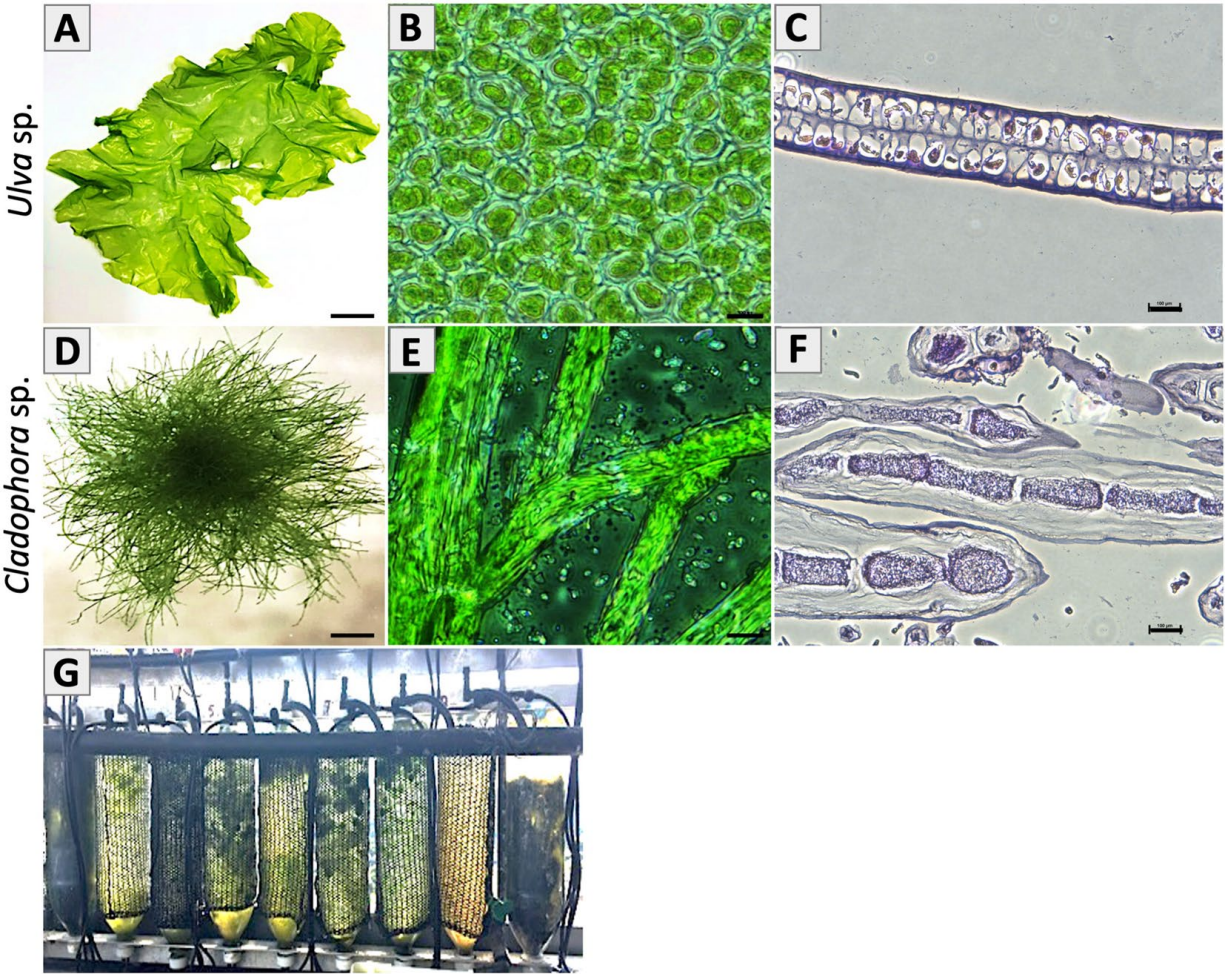
Marine green macroalgae: Thallus morphology macro view of (**A**) *Ulva* sp*.* and (**D**) *Cladophora* sp. Light microscopy observation (40×) of middle region reveals (**B**) *Ulva* sp. micro-porous structure and (**E**) *Cladophora* sp. branching fibrous filamentous structure. Hematoxylin and eosin (H&E) staining of cross-sections reveal tissue fragments of (**C**) *Ulva* sp. by-layer porous structure and of (**F**) *Cladophora* sp., fibers. (**G**) Macroalgae species cultivated in a Macroalgae Photo-Bioreactors (MPBR) system, design of Chemodanov, A., the Golberg Environmental Bioengineering Lab, Porter, Tel Aviv University. Cylindrical sleeve dimensions: 100 × 40 cm, thickness: 200 µm. *Scale Bars: (**A**) = 2.5 cm, (**B,E**) = 20 µm, (**C,F**) = 100 µm, (**D**) = 0.25 cm.

**Figure 2:**
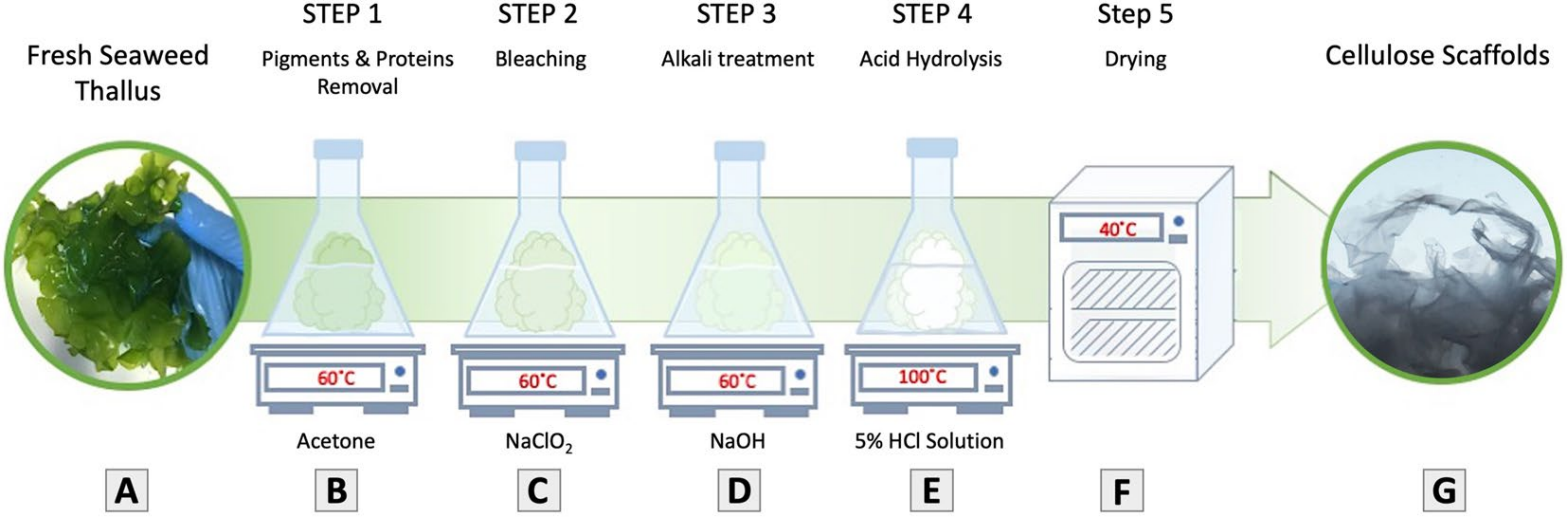
Scheme of decellularization treatment: cellular components are removed from a whole green macroalgae. (**A**) Fresh algae thallus samples were obtained, (**B**) soaked in acetate buffer to remove pigments and proteins (**C**) then soaked in bleach bath to remove polysaccharides of simpler structure than cellulose. (**D**) Following an alkali treatment with Sodium Hydroxide, to remove all excessive lipids and hemicellulose within the cell wall. (**E**) Further acid treatment is carried out with Hydrochloric acid, to remove all excessive polysaccharides, such as starch, that might remain close to the cell wall. Finally, the samples were rinsed in DW until reaching a neutral PH and obtaining a clear clean cellulose biomass. The samples were then filtered and dried making them a ready to be used acellular scaffolds.

**Figure 3:**
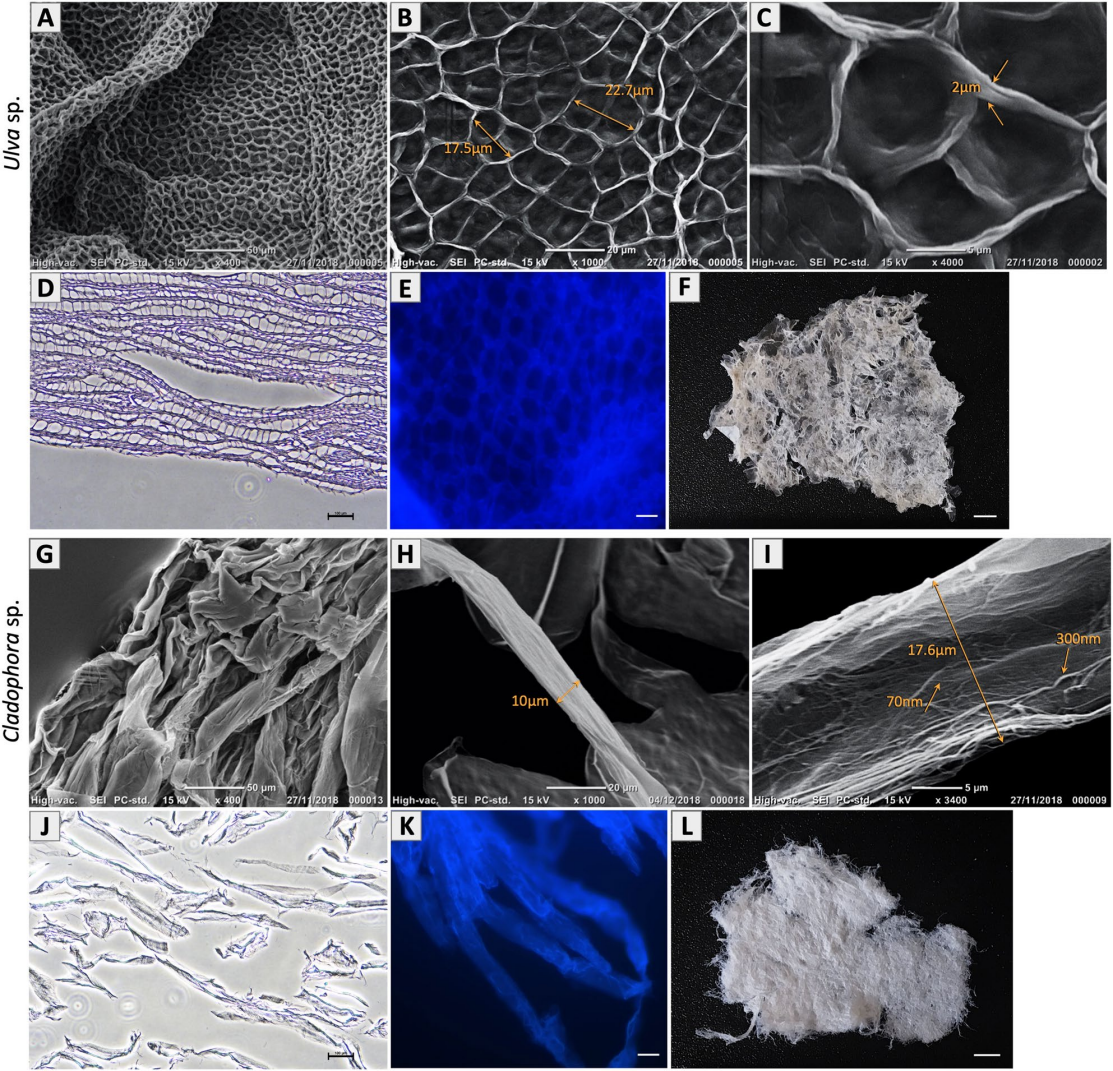
Decellularized seaweed cellulose: Structural surface area SEM imaging of (**A–C**) *Ulva* sp., acellular scaffold, show highly organized porous architecture with average pore size width 20.2 ± 4 µm (n = 50 analyzed regions) and cell wall thickness ranging between 0.5 and 2.0 µm (n = 10 analyzed regions). (**G–I**) *Cladophora* sp., acellular scaffold, show highly fibrous architecture with fiber diameter from 5 µm and above 80 µm (n = 55 analyzed regions), covered with microfibrils ranging in width between 55 and 400 nm (n = 50 analyzed regions). Hematoxylin and eosin (H&E) staining of cross-sections of decellularized scaffolds (**D**) *Ulva* sp. and (**J**) *Cladophora* sp., reveal eosin stain of the matrix and no hematoxylin (cell nucleus). Corresponding fluorescent microscopy images of seaweed cell wall stained with Calcofluor White, reveal middle region overview structural properties and confirm cellulose as the prime structural component of the seaweed scaffolds (**E**) *Ulva* spp. and (**K**) *Cladophora* sp. Both seaweed scaffolds were confirmed a-cellular, empty of cell organelles, indicating that the decellularization method was effective, and that the seaweed cellulose structural shape remained intact post decellularization treatment. Macro view of the decellularized seaweed (**F**) *Ulva* sp. and (**L**) *Cladophora* sp., were used as scaffolds for cell growth. *Scale bars: (**A,G**) = 50 µm, (**B,H**) = 20 µm, (**C,I**) = 5 µm, (**D,J**) = 100 µm, (**E,K**) = 10 µm, (**F,L**) = 0.25 cm**.**

Fresh macroalgae species *Ulva* sp. (Fig. 1A) and *Cladophora* sp. (Fig. 1D), were obtained, and examined for their structural composition variations, porous (Fig. 1B) and fibrous (Fig. 1E), respectively. Following, both species were decellularized (see “Materials and methods” section) (Fig. 2) to extract cellular content, obtaining a whole acellular natural seaweed scaffold. Observation analysis, including Scanning Electron Microscopy (SEM), fluorescent microscopy with Calcofluor White fluorescent dye that binds to cellulose, as well as histology analysis using Hematoxylin and Eosin (H&E) staining and DNA quantification test, were used to validate the decellularization treatment from both seaweed species, to determine cellulose explicit evidence, and to analyze both seaweeds structural composition variations (Fig. 3). SEM imaging of *Ulva* sp. (Fig. 3A–C) and *Cladophora* sp. (Fig. 3G–I) at different magnifications, revealed no remaining of cellular organelles or nuclei content in either of the seaweed scaffolds. H&E imaging of the decellularized algae samples (Fig. 3D,J), revealed the presence of eosin, which stained the cellular membrane in pink, and absence of hematoxylin, which stains cell nucleus in purple, in comparison to the H&E imaging of the fresh algae samples (Fig. 1C,F), which reveals cell nucleus. This confirmed both seaweed matrices a-cellular, emptied from their cellular components. However, it is important to note that the eosin in the *Ulva* sp. sample (Fig. 3D) was shown to be more distinct than that in the *Cladophora* sp. sample (Fig. 3J). This could be due to the different cell membrane of the two seaweed samples and the fragmentation caused by the cross-section methods. Furthermore, DNA quantification analysis (Fig. S1A) of the decellularized samples, reveal low DNA concentrations for the *Ulva* sp. and *Cladophora* sp., with 5.53 ± 2.80 ng/µl and 4.18 ± 0.35 ng/µl concentrations, respectively, confirming a-cellular. However, it is important to note that although DNA concentrations of fresh algae samples were higher than the decellularized samples, they obtained overall low values. The *Ulva* sp*.* with very low value of 9.59 ± 2.74 ng/µl, and the *Cladophora* sp. with higher values of 69.74 ± 16.50 ng/µl. This could be due to the low DNA content extracted from the *Ulva* sp. sample compared to the *Cladophora* sp. sample. This was confirmed with gel electrophoresis analysis (Fig. S1B), which validated high DNA content for the *Cladophora* sp. sample, yet very blurry results for the fresh *Ulva* sp. samples (see Fig. S1A,B).

The decellularized seaweed samples were further verified for their cellulose content using Calcofluor White fluorescent dye, which allowed for direct visualization of the stained cell wall with fluorescent microscopy and confirmed the presence of cellulose as the prime structural component of both seaweed scaffolds (Fig. 3E,K). All methods confirmed that the seaweed samples were acellular cellulose-based scaffolds, ready to be used as ECM, suitable for cell growth (Fig. 3F,L). For ease of reference, seaweed cellulose scaffolds will be referred to hereafter in terms of SC scaffolds***.***

### Seaweed matrices structural characterization

Post decellularization, samples of *Ulva* sp. and the *Cladophora* sp. scaffolds (Fig. 3F,L), were obtained for further analysis. Observation analysis confirmed the *Ulva* sp. and *Cladophora* sp. seaweed matrices' diverse structural compositions: porous and fibrous, respectively. Imaging of *Ulva* sp. matrix (Fig. 3A–E), revealed hollow cavities, organized in a comb-like network, with highly interconnected pores, while imaging of *Cladophora* sp. matrix (Fig. 3G–K) revealed entangled mesh, bundled fibrillar matrix. These were evident in all observational assessments, including SEM, H&E staining and Calcofluor White staining*.*

SEM imaging coupled with ImageJ software, enabled structural analysis and further understanding of the macroalgae acellular scaffolds’, including shape, size and surface morphology. SEM imaging of the *Ulva* sp. and the *Cladophora* sp. scaffolds were taken at different magnifications (Fig. 3). The *Ulva* sp. matrix was observed to have interconnected cellulose web-like polygonal pattern, with uniform pore size average width of 20.2 ± 4 µm dispersed along the matrix (Fig. 3B), and solid cell-wall ranging in width between 0.5 µm and up to 2 µm in the cell wall junctions (Fig. 3C), which confirmed a highly organized by-layer porous architecture and abundant surface area^24^. In comparison, imaging of the *Cladophora* sp. scaffold revealed a highly packed, threadlike filamentous matrix, composed of heterogeneous fibers, ranging in width from 5 µm and above 80 µm (Fig. 3G–I), overlaid with microfibrils ranging in width between 55 and 400 nm (Fig. 3I). However, due to the SEM metal coating we presume that the actual microfibrils diameters are even smaller. Furthermore, it should be noted that unlike the middle region shown with the SEM and Calcofluor White staining, the H&E staining, which reveal the cell wall membrane, stained with eosin, shows cross-sections perpendicular slices. Thus, these images do not reflect the true structure of both algae samples. While the *Ulva* sp. show some full-size pores, in some areas, the *Cladophora* sp. is fragmented. Therefore, it was impossible to reveal the whole fibrous structure and clearly confirm the samples size or width based on these images.

### Recellularization of seaweed cellulose scaffolds with mammalian cells

Observation analysis of the recellularized SC scaffolds enabled the evaluation of cell growth, cell morphology and biocompatibility. Readily sterilized scaffolds (1–2 mm^2^) were seeded with NIH3T3-GFP-actin fibroblast. The stable expression of actin-GFP by cells allowed us to follow the live cells cultured on the same scaffold at different time points from cell seeding and during the entire experiment.

**Figure 4:**
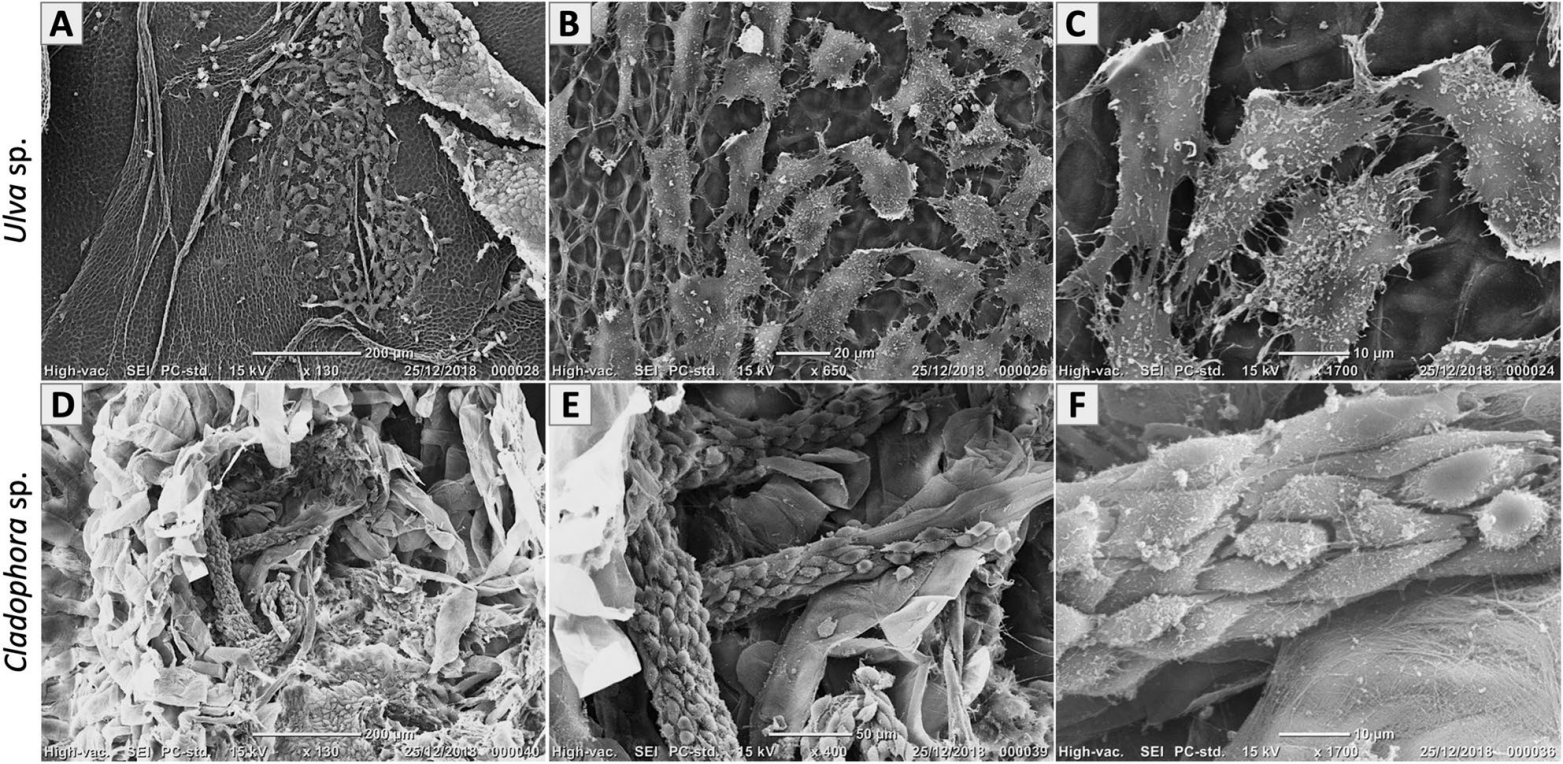
Recellularized seaweed cellulose scaffolds: SEM Imaging of sterilized cellulose scaffolds, recellularized with fibroblast after 4 weeks of seeding, reveal cell growth and cell attachments onto the (**A–C**) *Ulva* sp. porous matrix, with average cell size of 34.2 ± 8.4 µm (n = 40 analyzed regions), and along the (**D–F**) *Cladophora* sp. fibrous matrix, with average cell size of 20.1 ± 4 µm (n = 70 analyzed regions). Observations show elongated filament profusions (**C**) traced the *Ulva* sp. porous cell-wall matrix and (**D**) along the *Cladophora* sp. fibers, as well as connected to neighboring cells on both scaffolds’ surface areas. Both which confirmed cell-to-matrix and cell-to-cell interactions. *Scale bars: (**A,D**) = 200 µm, (**B,F**) = 20 µm, (**C**) = 10 µm, (**E**) = 50 µm.

SEM imaging analysis of both recellularized scaffolds, four weeks post seeding (Fig. 4), revealed a clear cell growth and cell attachments on the *Ulva* sp. porous scaffold (Fig. 4A–C) and on the *Cladophora* sp. fibrous scaffold (Fig. 4D–F)*.* Imaging revealed *Ulva* sp. scaffold overlaid with viable cells that adhered onto the surface area in random directions. Cells stretched across individual cavities and spread onto the porous matrix surface, while others, adhere to neighboring cells and formed continues layers. Fibroblast reached an average cell size of 34.2 ± 8.4 µm, on the *Ulva* sp. porous scaffold (Fig. 4B). Additionally, imaging showed cells filaments protrusions aligned along the matrix cell-walls, utilize the cellulose lattice as a backbone platform for attachment sites. Otherwise, observations showed elongated thin protrusions that traced the matrix cell-wall ridges and juncture-sites, as well as cells that formed connectivity towards neighboring cells (Fig. 4C). Whereas SEM imaging of the *Cladophora* sp. fibrous scaffold, exhibited cell attachments along the fiber’s axis with elongated spindle-like shaped morphologies (Fig. 4E). Cells were observed to reach an average cell size of 20.1 ± 4 µm on the *Cladophora* sp. fiber. The cells appeared to be fully attached to the scaffold’s fibers. The cells long axis was aligned parallel to individual *Cladophora* sp. fibers, covering the fibers’ surface area, with braid-like form, taking on the fibers’ shape (Fig. 4F). Additionally, SEM imaging showed cells connectivity with other cells along the fibers. While some areas of both scaffolds are seen unpopulated by cells, these observations confirmed cell-to-matrix and cell-to-cell interactions on both SC scaffolds. However, further investigation of cell growth on both SC scaffolds should take place.

**Figure 5:**
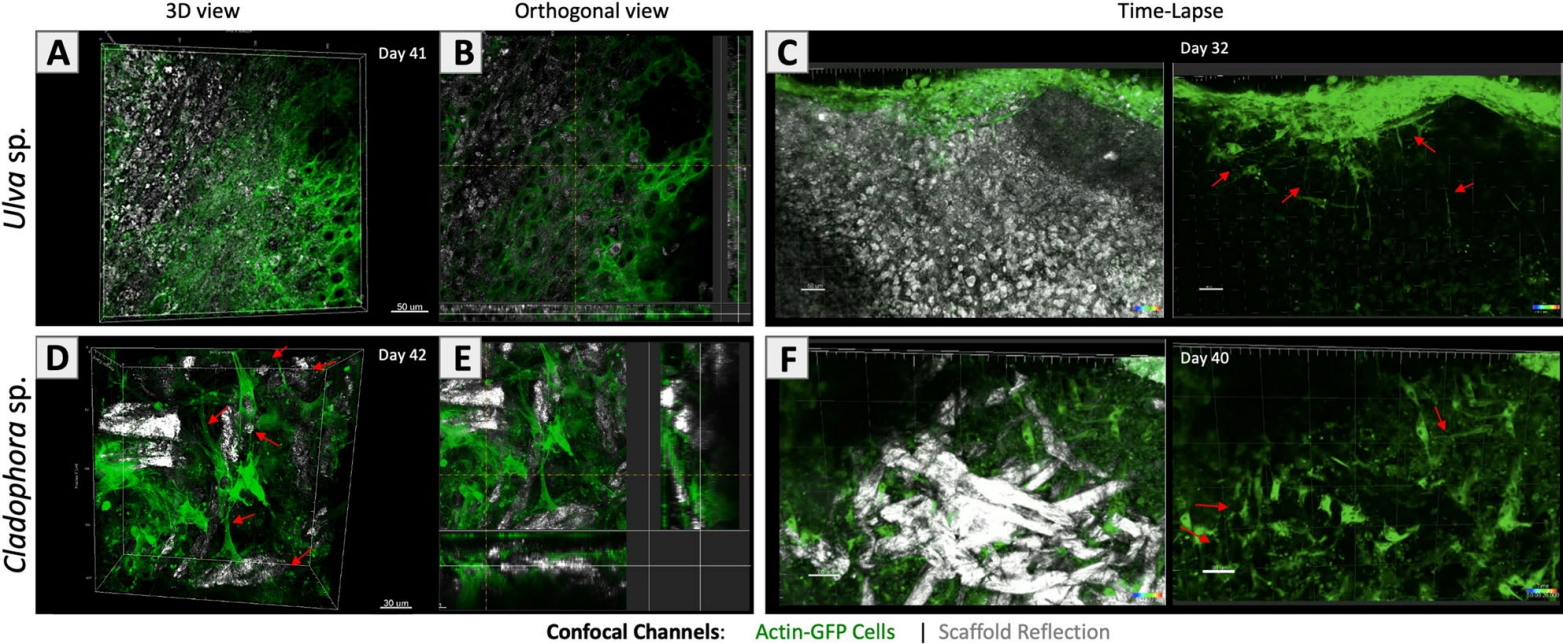
Cells growth on seaweed cellulose scaffolds: Fluorescence confocal microscopy imaging of live fibroblast (20 × 10^3^ cells/µl), labeled with actin-GFP (green), overlay the macroalgae cellulose scaffolds, detected in reflection mode. 3D Z-stack and orthogonal views (40×) reveal cell growth and attachments onto the (**A,B**) *Ulva* sp. porous matrix, (Day 41) and (**D,E**) *Cladophora* sp. fibrous matrix, (Day 42). Yellow dash lines indicate the location of the orthogonal cut. Additional time-lapse imaging (20×), available in Supplementary Movies S1–S4 reveal cell growth and spreading on the cellulose scaffolds (**C**) *Ulva* sp. (Day 32) and (**F**) *Cladophora* sp. (Day 40). Extended slender cell protrusions observed on both scaffolds, indicate that the cells remain alive and function during the entire experiment as they formed connectivity with neighboring cells and the scaffolds’ surface area. *Scale bars: (**A–C**) = 50 µm, (**D,E**) = 30 µm, (**F**) = 80 µm.

Confocal fluorescent imaging analysis of recellularized scaffolds enabled real-time monitoring and confirmed distinct cell growth, cell attachments and cell interactions onto both SC scaffolds. Shown here, 3D Z-stack and orthogonal confocal imaging of SC scaffolds *Ulva* sp. at day 41 (Fig. 5A,B) and *Cladophora* sp. at day 42 (Fig. 5D,E), recellularized with fibroblast (20 × 10^3^ cells/µl). The *Ulva* sp. scaffold’s surface area appeared to be covered with confluent monolayer cell formation, demonstrating cell spreading onto the porous matrix surface (Fig. 5A,B). While cells on the *Cladophora* sp. scaffold showed cells attached onto individual fibers, with stretched morphologies typically elongated in the direction of the fibers. Cells were also observed to interspersed between the fibrous mesh and bridge between the fibers (Fig. 5D,E*).* Additionally, confocal imaging time-lapse of the *Ulva* sp. and *Cladophora* sp. scaffolds were taken at day 32 and 40, respectively, post recellularization (Fig. 5C,F, Supplementary Movies S1–S4). The real-time imaging observations clearly show the formation of cell’s long slender protrusions within the porous *Ulva* sp. network (> 100 µm from nuclei center), and within the fibrous *Cladophora* sp. mesh, which verified cell spreading, attachments and migration within the SC scaffolds.

### Biocompatibility of seaweed cellulose assessment with alamarBlue assay

**Figure 6:**
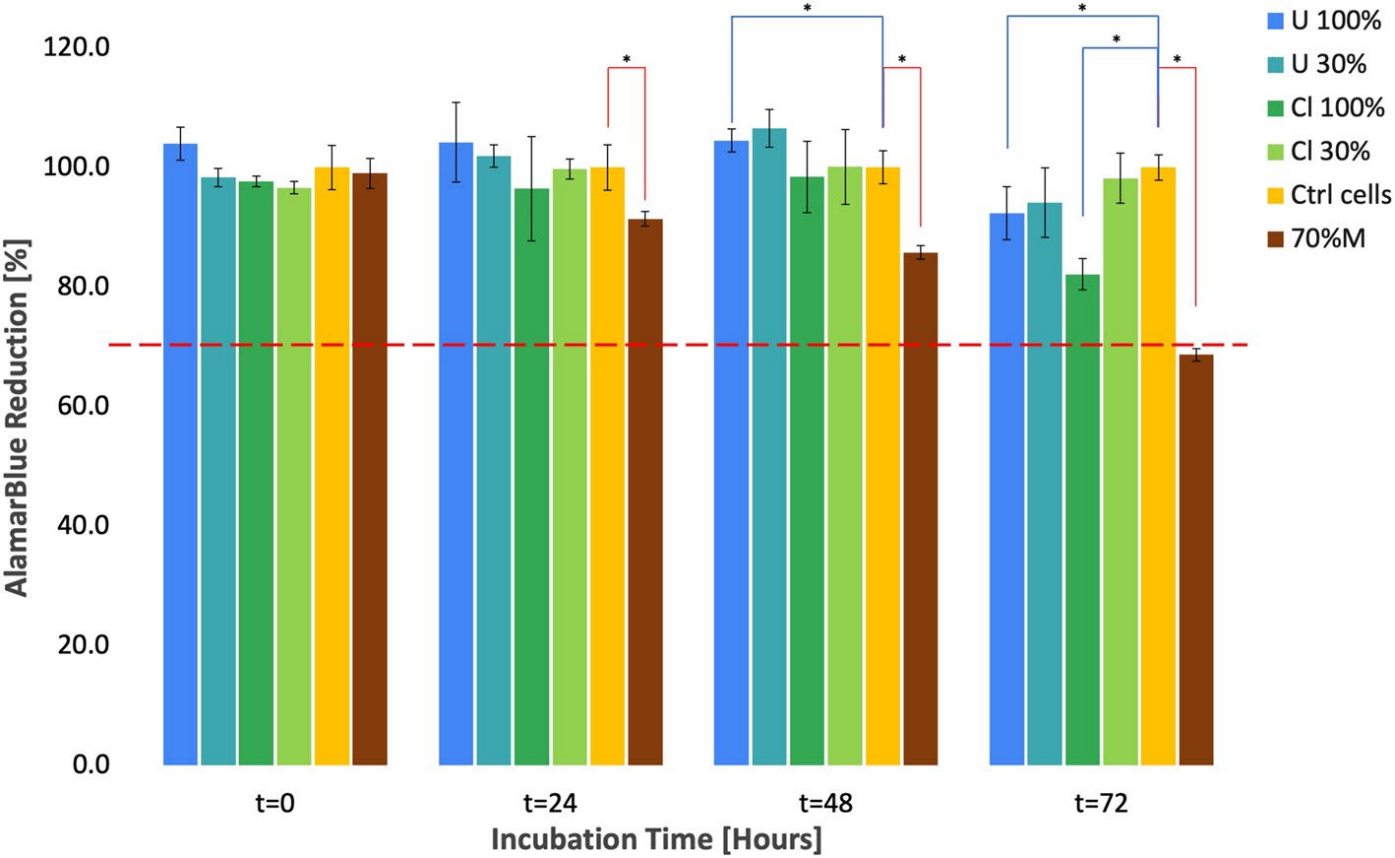
Cytotoxicity indirect test: SC scaffold *Ulva* sp. and *Cladophora* sp. were prepared according to ISO-10993. Cell viability was quantified with alamarBlue indirect test. Fibroblast incubated with 100% and 30% media extracted from *Ulva* sp. (U 100% and U 30%) and *Cladophora* sp. (Cl 100% and Cl 30%) cellulose scaffolds, at incubation time points t = 0, t = 24, t = 48 and t = 72. Control groups include negative control of cell culture incubated with regular media (Ctrl cells) and positive cytotoxic 70% methanol treatment (70%M). A red dashed line at 70% viability, distinguish between viable and toxic constructs. Values are expressed as mean ± SD, n = 5, *p < 0.05 (obtained by Student t-test).

**Figure 7:**
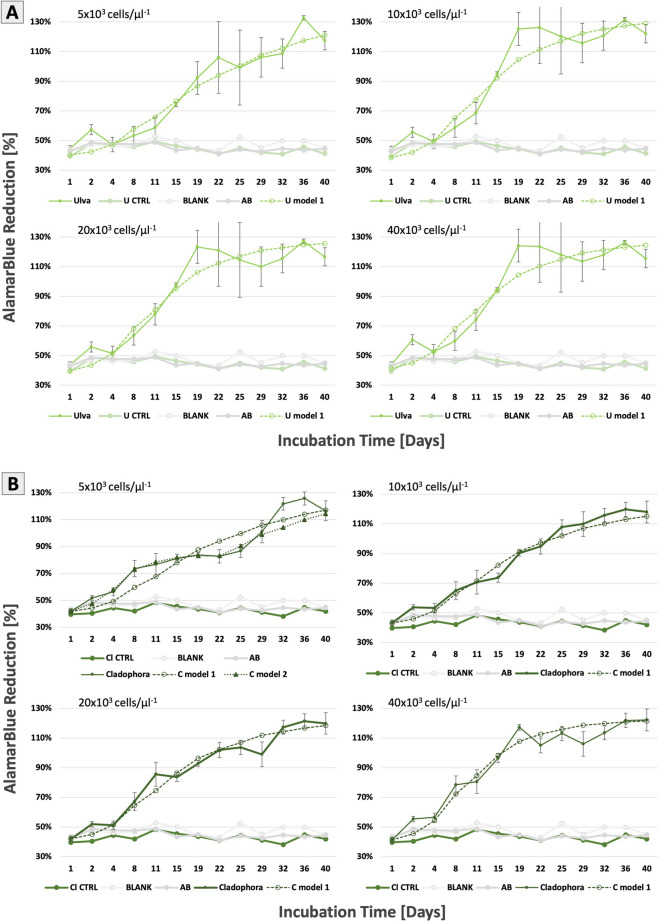
Cell viability direct test: Fibroblast seeded with seaweed cellulose scaffolds derived from (**A**) *Ulva* sp*.* and (**B**) *Cladophora* sp. at initial cell densities 5 × 10^3^, 10 × 10^3^, 20 × 10^3^ and 40 × 10^3^ cells/µl. The plots present cell growth over a period of 40 days for each cell concentration, relative to the alamarBlue percentage reduction. Control groups include *Ulva* sp. and *Cladophora* sp. scaffolds without cells, Blank media and 10% AB media solution. Values are expressed as mean ± SD, n = 3.

alamarBlue colorimetric assay was used to evaluate the biocompatibility of the cellulose macroalgae scaffolds *Ulva* sp. and *Cladophora* sp. by means of quantitative assessment of cytotoxicity, and consequently cell proliferation, with both direct exposure to the scaffolds and indirect extract method, according to the international ISO-10993 standards 5 and 12^25,26^, that are used for the biological evaluation of medical devices in animal testing and clinical trials. The main advantage of the alamarBlue (AB) method used in this study is that it is non-toxic to cells and does not require fixation, which enabled us a continuous monitoring and evaluation of live cell viability over a long period of time without sacrificing the cells as required in other methods, such as MTT, which is cytotoxic and could affect cellular morphology or cellular fate altogether^27^. Results for cell viability and cytotoxicity for both SC scaffolds are summarized in Figs. 6 and 7*.*

### Cytotoxicity evaluation of seaweed-cellulose scaffolds

The cytotoxicity for both cellulose-based macroalgae scaffolds, was determined by the indirect media extract method, applied to fibroblasts cultured in cell-culture dishes. The relative change of AB fluorescence signal, which directly reflects the metabolic activity of the cell culture, was evaluated after 24, 48 and 72 h incubation with 30% and 100% media extracts concentrations (Fig. 6). AB absorbance measurements show similar cell growth at the start of the experiment (t = 0) for all test and control groups (p > 0.05), with minor variations, due to the levels of cell coverage in each well. After 24, 48, and 72 h treatment, high AB reduction measurements above 80% were recorded for both the for the *Ulva* sp. and *Cladophora* sp. scaffolds and for both 30% and 100% media extract. These results showing more than the standard 70% viability, confirmed the non-toxicity of both SC scaffolds.

### Cell viability evaluation with seaweed cellulose scaffolds

The AB assay enabled us to monitor live cell viability cultured on both SC scaffolds over a period of 40 days. The evaluation of cell growth with direct contact was determined by the relative increase of AB fluorescence signal over time, correlated to cell proliferation, in accordance with the AB assay, at four cell concentrations for each scaffold (Fig. 7). Results demonstrated that both the porous *Ulva* sp. and the fibrous *Cladophora* sp. matrices, supported a long-term cell growth, indicated by an overall increase of AB reduction percentage with an average positive upward trend of 2.7-fold, with variation trends for both scaffolds. Cell viability on the *Cladophora* sp. scaffold showed a consistent and steady increase overtime (Fig. 7B), while cell growth on the *Ulva* sp. scaffold started with a steeper upward trend until week 3, followed by a stable plateau saturation level (Fig. 7A). However, it is important to note that although the seeded SC scaffolds were transferred to non-treated plates for the entire experiment, this method does not assure 100% accuracy detecting only the viability of cells on the SC scaffold alone.

Parametric Student’s T-test comparisons coupled with Fischer Combined Probability test, show a highly significant difference (combined p < 0.0001) between the *Ulva* sp. and *Cladophora* sp. scaffold test groups, for all four cell concentrations, as well as between the scaffolds’ test and control groups. The viability results for all control-groups of the SC scaffolds without cells, show no significant difference, with a stable AB percentage reduction mean of 45% ± 2.

More specifically, the plots at week one revealed a higher cell proliferation within the *Cladophora* sp. scaffold, with 71% ± 6.15 average percentage reduction for all cell densities, compared to 58.8% ± 4.18 for the *Ulva* sp., while cells on the *Ulva* sp. scaffold reached a higher proliferation from week 2 onwards (> 90% ± 10.73) for all cell densities, compared to the *Cladophora* sp. scaffold (83.8% ± 9.5).

### Cell proliferation rate increased in correlation to cell concentration

**Figure 8:**
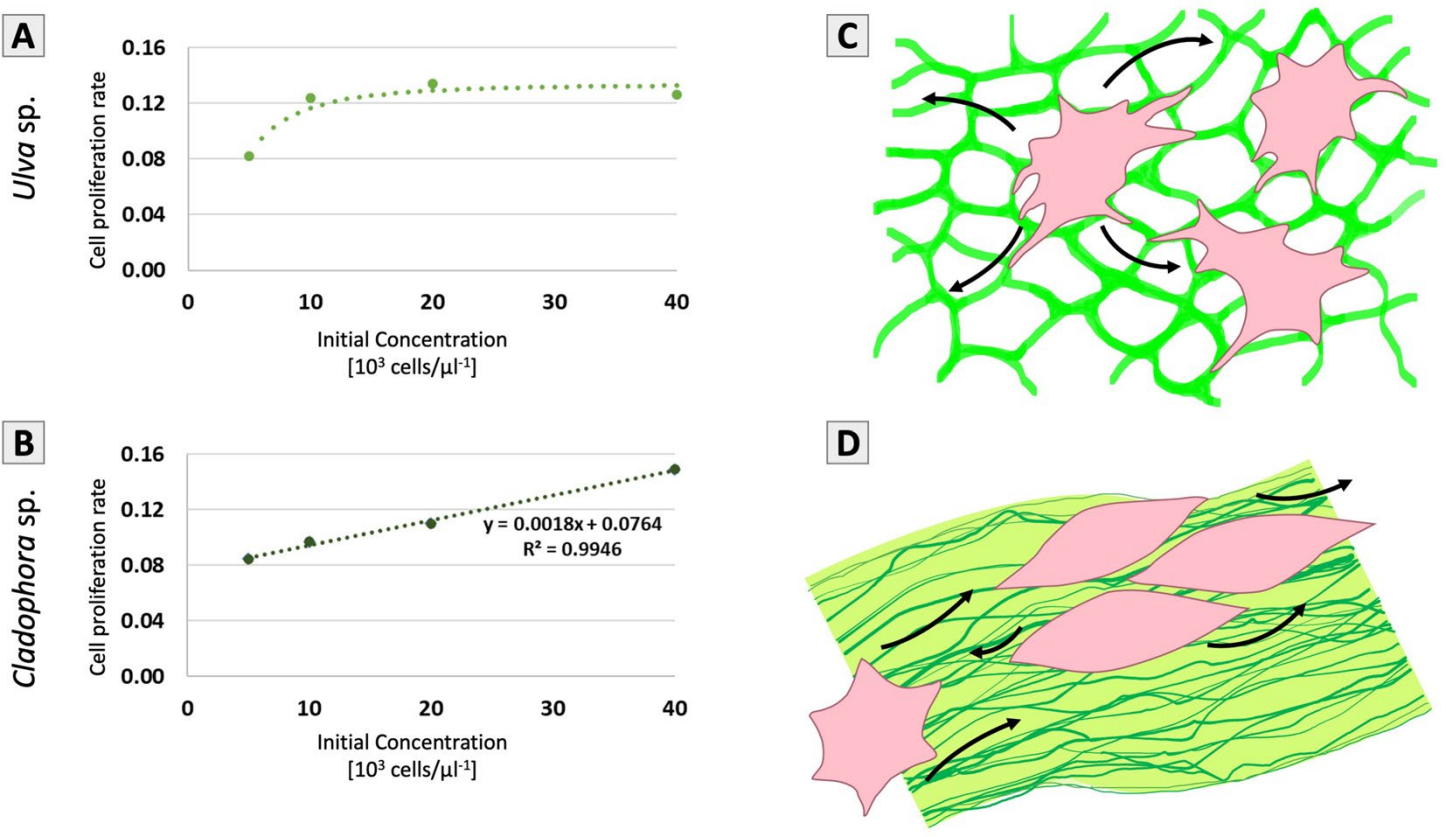
Cell growth in correlation to cell concentrations: Modeled cell proliferation rates as a function of initial cell seeding concentration for the (**A**) *Ulva* sp., which reached rapid increase following by cell saturation at high cell concentrations and for the (**B**) *Cladophora* sp. with a linear increase correlated to cell proliferation rate and initial cell seeding concentrations. The plots present cell growth upward trends at 5 × 10^3^, 10 × 10^3^, 20 × 10^3^ and 40 × 10^3^ cells/µl cell concentrations. Scheme of cell migration and alignment in correlation to SC structures: the (**C**) *Ulva* sp. matrix facilitates migration opportunities in all directions, which results in rapid cell growth, as cells ‘cover’ the scaffold’s microporous surface area, following proliferation rate decrease, due to early cell saturation. The (**D**) *Cladophora* sp. structure facilitates migration opportunities along the fiber elongated axis, guided by microfibrils that overlay the fiber’s surface, which results in linear increase of proliferation rates.

A logistic growth model, used to estimate cell proliferation rates in the different experiments, was fitted to the results from the viability tests, using Eq. (3). Cell proliferation rates (r) were calculated for each SC scaffold type and initial cell concentration ($C_{i}$) by fitting a proliferation model to data points of AB percentage reduction measured throughout the experiment. The prediction models, which obtained a Root Mean Square Relative Error (RMSRE) of 0.077 ± 0.007 for the *Ulva* sp. scaffold and 0.077 ± 0.018 for the *Cladophora sp*. scaffold, were incorporated into Fig. 7 (dashed lines). Cell proliferation on the *Ulva sp.* scaffold was unstable during the first few days (lag period), therefore its $t_{0}$ was set to the fifth day of the experiment (day 4). Cell proliferation on the *Cladophora sp.* scaffold was stable from the beginning, and thus its $t_{0}$ was set to the time of the first measurement (day 1). Next, cell proliferation rates for both scaffold types were plotted as a function of initial cell concentration (Fig. 8A,B). Proliferation rate in the lowest initial cell concentration (5 × 10^3^ cells/µl) were similar for both scaffolds (r = 0.08). However, the rate of cell proliferation on the *Cladophora* scaffold, increased linearly with initial cell concentrations (R^2^ = 0.995), whereas the rate of cell proliferation on the *Ulva* scaffold, as a function of initial cell concentration, could be described as a second order Hill equation ($r = 0.134\frac{{C_{i}^{2} }}{{C_{i}^{2} + 3.88^{2} }}$, RMSRE = 0.043), leveling off at an initial cell concentration of 10 × 10^3^ cells/µl. In summary, the model exhibited that in the examined range initial cell concentrations affect proliferation rate differently on each SC scaffold type, following a second order Hill function on the *Ulva* sp. scaffold and a linear trend on the *Cladophora* sp. scaffold.

## Discussion

In this work we suggest novel cellulose scaffolds derived from marine green macroalgae species *Ulva* sp. and *Cladophora* sp. The cellulose scaffolds were extracted and analyzed for their structural variations and biocompatibility in vitro. The structural-cellular interactions between the two seaweed cellulose scaffolds and NIH3T3 cells, were examined and reported in this study.

Key considerations for selecting a suitable scaffold, when designing a bioartificial ECM environment, are its biocompatibility and ability to support cell growth and viability over time. Many natural and synthetic biomaterials are suitable resources for cell growth in tissue engineering. However, there is still an ongoing search for alternative, inexpensive matrices that could replace native tissue permanently^1^. In recent years cellulose-based matrices have ignited novel bio-based scaffold fabrication^3,7–9^. However, seaweed cellulose is still poorly investigated. Cellulose biopolymers from marine resources are attractive biomaterials, due to their little to none toxic reactions, and natural antimicrobial bioactive compounds^28^, relatively low cultivation and production cost^17^, as well as, minor or absence of lignin content, and sustainable biostable features^4^, which are appealing for applications that require no degradability and no conductivity as reinforcement, or as inert, composite biomaterials.

Decellularization could be achieved through numerous methods, including mechanical and enzymatic approaches^29^. However, in order to achieve the best results to decellularize seaweed, while preserving structural composition intact, it was essential to fully decellularized a whole seaweed tissue from its cell content yet sustain undamaged cell wall. Following acid hydrolysis decellularization approach^30,31^, and its optimization for a whole tissue sample (Fig. 2), the removal of all cellular content from the macroalgae cell wall was achieved. SEM imaging analysis were conducted to confirm the decellularization approach and to ensure that the acellular scaffolds maintained their core structure after the decellularization treatment. SEM imaging (Fig. 3A–F) of both seaweed matrices, confirmed an acellular, intact structural shape, obtaining the original tissue emptied from its cell content. Additionally, cellulose content was validated as the main cell-wall component for both SC scaffolds, *Ulva* sp. and *Cladophora* sp., with Calcofluor White fluorescent dye (Fig. 3E,K), which has been proved to be an effective method for a simple and quick cellulose detection in plant tissues^32^. These findings were consistent with previous studies of the two macroalgae species^14,24^.

It is worth noting that utilizing strong chemicals for the removal of cell content and the isolation of cellulose has indeed proven effective, however future optimization of the decellularization treatment is necessary in order to reduce or use no chemicals while promoting an economically and environmentally green approach. For example, pulsed electric field (PEF) has been previously studied^33^, and shown to be effective, thus could be applied to decellularize SC, as well as sporulation inhibitors extraction^34^, that could further be explored to decellularize SC. Additionally, integrated process^31^ over direct cellulose extraction process, can promote sustainable biorefinery design approach, for cellulose production with minimum environmental impact.

An additional key factor for selecting a suitable scaffold is its structural properties. On one hand scaffolds are required to advance cell growth, while providing structural and mechanical support for cell attachments on the ECM binding sites^35–37^, and on the other hand they promote permeability to ensure the diffusion and transport of nutrient, cell signaling, oxygen, and growth factors^38^, which in turn impact cell fate^39^.

Previous studies have shown direct correlation between scaffolds structural properties and cells behavior^40,41^. In this research, macroalgae *Ulva* sp. and *Cladophora* sp., have demonstrated distinct cellulose variations: porous and fibrous, respectively. Thus, we hypothesized that variations of the SC scaffolds’ structural morphologies, surface topographies and boundaries of the overall surface area (fiber width, porous tissue) enabled or limited cell attachments, cell spreading and migration orientations, and as a result influenced distinctly the fibroblasts cell growth, proliferation and morphologies.

For example, in porous scaffolds, different pore size could directly promote or hinder cell functionality^35,41^, thus ECMs with different pore sizes could be optimal for various tissue engineering applications^41^. In comparison to other cellulose derived porous scaffolds, the *Ulva* sp. SC observed in this study consist of an intermediate pore size (10–30 µm) (Fig. 3B), which is larger than bacterial nanocellulose (BCN) pore size 1.66–98.7 nm^10^ (defined as the space between the BCN nanofibers), while smaller than terrestrial plant-based cellulose, for example apple, carrots and celery with pore sizes that ranges between 70 and 420 µm^9^, and is also smaller than custom collagen sponges (50–200 µm), such as the BioMatrix (SpongeCol). Scaffolds with various pore size (50–350 µm), were described as macroporous, with pore sizes that exceed the cell size. Macroporous scaffolds are shown to promote cellular infiltration into the pores^42^ and support adherence to the flat surface area around the cavities, or onto the pore walls^43^, and thus increase 3D cellular organization. While microporous scaffolds with pore size (0.1–10 µm) that are smaller than the cell size^42^, limit cell invasion into the pores, and rather promote contact to the pore margins^44^. Ultimately spreading onto the surface area, creating cell-to-cell interactions and forming a continues sheet onto the scaffolds’ surface area^42^. For instance, MSC cells cultured on large pore size (> 100 µm), displayed elongated stretched morphologies along the cell wall, while cells cultured on smaller pore size (< 50 µm) displayed more oval-shaped morphologies with attachments in three-dimensions stretched across the pores^43^. In comparison, fibroblast, cultured on the *Ulva* sp. intermediate pore size scaffolds (Fig. 4A–C), displayed polygonal-stretched appearance, with cells size (34.2 ± 8.4 µm) exceeding the average pore size (20.2 ± 4 µm). SEM imaging revealed 2D cellular organizations of individual cells spread onto the SC surface, which initiated interactions with neighboring cells, while others formed monolayer ‘sheets’ onto the *Ulva* sp. surface area (Fig. 4A).

These findings are consistent with previous studies^21,42^ and with the confocal imaging findings, conducted separately from the SEM imaging testing, here too the confocal imaging confirmed monolayer cell growth appearance (Fig. 5A). Moreover, the confocal imaging revealed elongated filaments protrusion that extended towards the matrix surface area, as well as through and in between the cavities, which were apparent in the GFP labeled actin stress fibers (Fig. 5A,C), demonstrating cell-to-ECM interactions.

Consisting of high interconnected porous morphology and a distinctive intermediate pore size, we suggest that the *Ulva* sp. SC scaffold in this study, could provide a dynamic surface topography with abundant and evenly dispersed, attachment sites for continues cell growth, and spreading, and thus could impact cell migration directionality in more random orientation (Fig. 8C). These finding were consistent with previous studies of cell growth on flat 2D surfaces as well as 3D models with small porosity, which are characterized with flat and stretched monolayer morphologies, random growth directionality and good cell–surface interactions. Similar porous ECMs, were also found to be advantageous for differentiation, cell proliferation, cell viability, cell–cell and cell–ECM interactions^40^, favorable to endothelial and dermal cells^41^.

In comparison, fibers’ properties in fibrous scaffolds, too were shown to have significant impact on cell fate^38^. The *Cladophora* sp. observed in this study, obtain high fibrous matrix with a versatile fiber diameter (5–80 µm) (Fig. 3G–I), ranging from macroscale fibers that are found in plant cellulose, such as banana, sisal and coir (30–300 µm)^45^, to nanoscale microfibrils found in maze, cotton, celery and *Arabidopsis thaliana *(1–25 nm)^46^. Moreover, studies have shown that nanofibers enhance cell attachment and proliferation and effect cell spreading^38,47–49^. The microfibrils that overlay the *Cladophora* sp. fibers (Fig. 3I), were found to have a wide range of diameter width (40–400 nm), consistent with those found in nanocellulose, derived from various sources, from bacteria nanocellulose (BCN) fibrils, (10–30 nm)^4,50^*,* and lignocellulosic resources (< 150 nm)^51^ to synthetic electrospun microfibrils (< 500 nm)^52^, fibrillar electrospun collagens (50–300 nm)^53^, and interestingly, in comparison with nontopographic grooved surfaces, ranging between 330 and 2100 nm, groove widths^54^.

SEM imaging of fibroblasts cultured on *Cladophora* sp. scaffold displayed spindle-shaped elongated morphologies, with cell size (20.1 ± 4 µm) smaller than the average fiber diameter (38.1 ± 34 µm), and the cell’s long axis appeared to be aligned parallel to the *Cladophora* sp. fibers (Fig. 4D–F). These growth patterns are consistent with cell morphologies found in native 3D fibrous tissue structures, as well as on topographical or grooved surfaces^40,55^, which have demonstrated high influence on cell behavior, including the orientation, morphologies and proliferation of cells by geometrical cues, associated with contact guidance^54,56^. Thus, we suggest that the high fibrillar surface topography, visible on the *Cladophora* sp. fibers (Fig. 3F)*,* could affect contact guidance developments and therefore, enhance cell attachments and elongated morphologies along the fibers, as well as guide cell spreading and migration directionality onto the fiber axis (Figs. 4F, 8D).

Additionally, highly entangled matrices were shown to promote permeability, that advance cell survival, growth opportunities and cell attachments within the mesh layout, and bridge gaps between nearby fibers^49^. Consisting of high entangled fibrous morphology, versatile fiber diameter and nanofibrils overlay, the *Cladophora* sp. SC scaffold in this study, could provide with abundant topographical cues, for attachments and spreading along the fiber, and thus greatly contribute to the formation of connectivity between the cells as they attach onto the scaffold’s fibers, and establish cell-fiber contacts, as well as cell-to-cell interactions, (Figs. 4F, 5B), which impact cell growth, proliferation and cell migration orientation in one dimension (1D) along the fiber axis, as well as the formation of elongated filament protrusions between the fibers (Fig. 5D).

Similar to other cellulose derived biomaterials^3^, these porous and fibrous seaweed cellulose models offer the necessary structural properties to support different cell types in numerous tissue engineering applications. For example, *Ulva* sp. intermediate pore size and *Cladophora* sp. fiber dimension could support mammalian dermal cells, and are suitable for drug testing, skin and wound healing applications^35,40^. Thus, both seaweed structural properties could serve as an effective ECM when utilized as scaffolds for cell growth and have shown correlation to cell behavior with significant impact on cell morphology, attachments, and motility. It should be noted however, that cell growth and cell spreading in this study were shown to favor some areas of the scaffolds, while evade other areas (Fig. 4A,D), which could be attributed to the seeding technique. However, these findings, including cell dynamics and cell coverage on SC scaffold surface area, should further be investigated.

Another key consideration for selecting a suitable scaffold is biocompatibility, which ensures cell viability, proliferation, cellular attachments, and differentiation. In this study, the alamarBlue (AB) assay, enabled both the monitoring of live cell viability, with direct contact test, over a long period of time without scarifying the cell culture, and the evaluation of cytotoxicity and cell viability with media extracts, indirect contact test. Both SC scaffolds demonstrated to be nontoxic, with 7.6% and 17.8% loss of metabolic activity, after 72 h incubation in 100% media extracts for the *Ulva* sp. and *Cladophora* sp., respectively, (p < 0.05), while maintaining a constant high viability in the presence of 30% media extract (p > 0.05) (Fig. 6). Despite the reduction in cell viability, when exposed to 100% SC scaffolds media extracts for 72 h, cell viability above 70% is considered to be non-toxic in accordance to ISO 10993 standard, and was consistent with other studies^57–59^. The cell viability decrease could be attributed to the adherence of protein from the media extract onto the SC scaffolds during incubation, as suggested in previous studies with collagen scaffolds^57^.

In addition, cell viability analysis was evaluated through direct fibroblasts seeding, at various cell concentrations, onto the *Ulva* sp. and *Cladophora* sp. SC scaffolds. It should be pointed out that the SC matrices in this study were not coated nor cross-linked with any additional reagents such as ECM proteins, which have been utilized in other studies, to enhance cell attachments prior to cell seeding^7^. Cell viability for both SC scaffolds and all four concentrations, increased with an average positive upward trend of 2.7-fold during the experiment. These results are consistent with previous studies of viability tests that used AB with plant cellulose^9,60^ and marine collagen^57^. Furthermore, the upward viability trends in this study, showed a significant difference for the two SC scaffolds, with a combined p < 0.0001 for all four cell concentrations.

However, differences in cell viability between the two scaffolds, and between cell concentrations, could be attributed to numerous reasons, including cell growth rate correlated to initial cell seeding efficiency, matrices permeability and exposure area, which impact cell fate opportunities. As well as, the SC scaffold structural properties (porous and fibrous), which offer advantages and disadvantages to cell growth and to cell-media-scaffold interactions, contact guidance, which orient cell attachments, and the overall shape of the scaffold, which provides boundaries for cell spreading and orientation. Thus, we propose that the two SC matrices structural properties and surface area that could be occupied by cells, provide a unique framework for cell growth and therefore impact cell-to-cell interactions differently, which suggests the correlation between scaffold structural geometry and topography to cell fate and functionality. The *Ulva* sp. microporous scaffold enabled cell-to-cell interactions in all directions onto its surface area (Fig. 4A–C), advancing cell proliferation in all surface directions, in two dimensions (Fig. 8C). While, in comparison, cell spreading on the *Cladophora* sp. scaffold, was limited by the fibers’ width, and guided by the overlaid microfibrils (Fig. 4D–F), advancing cell-to-cell interactions in one dimension, along the fiber elongated axis (Fig. 8D).

In addition to the observational analysis, cell proliferation onto the *Cladophora* sp. scaffold is supported also by the model, presenting a linear increase in proliferation rate as a function of initial cell seeding concentrations (Fig. 8B). Thus, we hypothesized that the initial seeding concentration and the SC matrix structural surface area could determine the number of fibers along which proliferation occurs, and consequently impact the growth rate.

In contrast, cell proliferation on the *Ulva* sp. scaffold structural surface area (Fig. 8C), showed a slower proliferation rate in low concentrations, yet accelerated rapidly as concentrations increased (Fig. 8A). The Hill function, presented in this study, is commonly used to describe the relationship between the concentration of free ligand and the fraction of receptors bound by ligands^61^. Thereby, we simulated the concentration of free ligands to free “migration opportunities”. Thus, in a two dimension structures we could obtain a second order Hill function, in which cell growth has more “migration opportunities” in random directions, and as a result, the growth rate increases more rapidly, while cells ‘fill’ the scaffold’s surface area, rates decreases due to saturation effects (Fig. 8A,C). The structural features of each SC scaffold, differing cell alignments, facilitated cell migration, occupied the scaffold surface area in linear or all directions, which in return impact cell proliferation. Therefore, selective cell types on SC structures, could be highly advantage on the development of implanted devices^35,40,41^.

In summary, this study proposes the simple production of novel biomaterial from two seaweed cellulose structures through a simple decellularization-recellularization approach. Seaweed cellulose cultured with 3T3/GFP-actin enabled real-time evaluation of cell growth. The two seaweed *Ulva* sp. and *Cladophora* sp. porous and fibrous structural composition variations, enabled a simple model for the comparison of cell behavior. Biocompatibility analysis showed an overall upward trend of fibroblasts proliferation at all four cell densities for both SC scaffolds. Cells, on both structural scaffolds, were observed to obtain a high percentage of viability over a period of 40 days, proposing cellulose macroalgae as a highly compatible scaffold to support cell growth over a long period of time.

### Future study and limitations

The disparity results between the scaffolds could support our estimations of cell growth and cell behavior as influenced by the SC scaffolds structural properties, including morphology and topography. While the fibrous *Cladophora* sp. scaffold could benefit cell seeding at initial attachments, or at high cell concentrations, the microporous *Ulva* sp. scaffold could benefit cell growth over time and at lower cell concentrations. Thus, proliferation activity of cells could be altered specifically to desired cell type and applications, including bioartificial tissues, wound dressing and encapsulations, which are not subjected to biodegradability, or as a natural scaffold for the growing cultured meat industry. Furthermore, diverse fabrication and drying methods, including freeze dry and 3D printing, will allow to alter the SC scaffolds shapes, as desired for specific applications. Our in vitro model indicated that both SC matrices could offer a natural structural support and provide a biocompatible template stimulus to guide cell proliferation and tissue formation, without causing toxic effect to mammalian cells, while still environmentally produced and used. However, it is necessary to conduct further in vivo biocompatibility evaluation of both SC scaffolds to better understand the interactions and affinity of seaweed cellulose biomaterials with mammalian cell growth and tissue. Furthermore, the study of scaffolds’ biostability, degradation and micro-mechanical performance with biological interfaces, as well as continues cell dynamics, including scaffold area coverage, and the secretion of newly ECM depositions by cells, are significant in future studies in order to provide more in-depth analysis for the long-term SC implant survival and efficacy.

## Materials and methods

### Preparation of materials

Green marine macroalgae species *Ulva* sp. and *Cladophora* sp. were used in this work as a model for their structural composition variation: a porous and a fibrous matrix structure, respectively (Fig. 1A–F). These two species, which have worldwide distribution, are found in the intertidal and shallow waters of the Israeli Mediterranean seashores. *Ulva* sp. and *Cladophora* sp. are known for their fast growth rates^17^, and are considered as potential feedstock for biorefineries^16,33^. *Cladophora* sp. was cultivated under controlled conditions using cylindrical, sleeve-like macroalgae photo-bioreactors (MPBR, Polytiv, Israel), with sleeve dimensions of 100 cm length, 200 μm thickness, 40 cm width, and total circulation volume of 3400 l seawater (salinity 3.9%, pH 8.2)^16^ (Fig. 1G). *Ulva* sp. was obtained from the seaweed unit of Israel Oceanographic & Limnological Research, Haifa, Israel (IOLR) and Tel Aviv University. Collected biomass from Haifa IOLR was transported to the laboratory in plastic bags filled with seawater. All samples were cleaned, sorted manually to get clean monocultures and documented for their morphology and histology.

### Seaweed cellulose decellularization

A whole organ or tissue decellularization approach is a process that is used to isolate the extracellular matrix (ECM) of a tissue from its inhabiting cells, leaving a “ghost” ECM scaffold of the original tissue^62^. Following an efficient decellularization treatment^30,31^ and its optimization for a whole tissue culture, cellular content was extracted from the two macroalgae species *Ulva* sp. and *Cladophora* sp. (Fig. 2). Fresh algae biomass samples were obtained, cleaned and sorted by hand (Fig. 2A). 100 g wet weight *Ulva* sp. and *Cladophora* sp. seaweed samples, were boiled in acetone bath (20% w/v) at 60 °C for 60 min, repeatedly 4 times, in order to remove pigments (chlorophyll) and proteins (Fig. 2B). Residual biomass was boiled in acetate buffer bath, containing 1.17 g Sodium Chlorite (NaClO2) (20% w/v), at 60 °C for 6–8 h, spurring bleaching and the removal of simpler structure polysaccharides (Fig. 2C). The bleached seaweed residues were pH neutralized by washing with distilled water, and then alkylated in 0.5 M Sodium Hydroxide (NaOH) bath (20% w/v), at 60 °C for 8–10 h, to remove all excessive lipids (Fig. 2D). Following the alkali treatment, the seaweed residues were pH neutralized by washing with distilled water, and then acidified in a hydrochloric acid (HCI) (5% v/v), at 100 °C for 10 min (20% w/v), or until boiling started (Fig. 2E). Next, samples were rested overnight at room temperature to remove all excessive polysaccharides that might remain close to the cell wall. Finally, the samples were carefully rinsed repeatedly in DW, until reaching a neutral pH (SevenExcellence pH Meter).

### Seaweed cellulose scaffold fabrication

Obtaining a clear clean cellulose biomass, the seaweed residues were then filtered and dried at 40 °C in an oven for 24 to 48 h, or at room temperature (RT°C), on a flat surface for a period of 4 to 7 days (Fig. 2F), obtaining a final whole-tissue cellulose scaffold, ready to be used for cell growth (Fig. 2G). Using a digital caliper (Holex), *Ulva* sp. and *Cladophora* sp. scaffolds were measured for their thickness, 0.1 mm and 0.15 mm, respectively (Fig. 3F,L), and for their area dimensions for each experiment. Decellularized samples with area dimensions that range between 1 and 2 mm^2^ were used for observation imaging analysis. Scaffolds for the biocompatibility tests were fabricated with specific dimension area for the viability direct test (uniformed 2 mm^2^ circles) and cytotoxicity indirect test (6 cm^2^ per 1 ml) as described below. Samples post-decellularization treatment were analyzed using fluorescent microscopy observation with Calcofluor White staining, Scanning Electron Microscopy (SEM) observations, H&E staining and DNA quantification, as described below.

### Cellulose determination

To determine the presence of cellulose in the decellularized scaffolds, fluorescence staining solution consisting of Calcofluor White reagent (Ref. 18909; Sigma-Aldrich), which binds to cellulose in the plant cell wall, and 10% potassium hydroxide (KOH) (Ref. P5958; Sigma-Aldrich) (1:1) was used. The Calcofluor White fluorescent dye solution was deposited directly onto the seaweed decellularized samples, which were placed onto glass slides. Fluorescence Microscopy was used to observe the samples. The Evans blue present in the stain, emits fluorescence at a wavelength of 395–415 nm and permits a rapid visualization of cellulose presence in the decellularized seaweed cell wall (Fig. 3E,K).

### Seaweed cellulose scaffold histology

To evaluate and analyze the decellularized seaweed cellulose scaffolds, *Ulva* sp. and *Cladophora* sp. fresh and decellularized samples were embedded in paraffin and sectioned into 4 μm thick slices perpendicular to the surface. The sections were mounted on glass slides (4 sections per slides), stained with hematoxylin and eosin (H&E) reagent (Patholab, IL) and visualized under an optical microscope (Nikon Eclipse TS2, Japan). All image processing was performed with ImageJ software (ImageJ v. 1.51, NIH).

### DNA quantification

The evaluation of acellular scaffold, emptied from its cellular organelles post decellularization, were further determined using plant genomic DNA concentration and purification analysis (Thermo Scientific GeneJET # K0791). The concentration was measured with a NanoDrop spectrophotometer (ND-2000, Thermo Scientific), used for a quick and simple wavelength absorbance analysis. Fresh and decellularized *Ulva* sp. and *Cladophora* sp. samples were examined (n = 3 for each sample). Wavelength absorbance of all samples (1 μl solvent) were compared with blank sample and purified DNA sample with a nucleic acid to protein (A260/280) indicator and ratio between 1.7 and 1.9. Furthermore, gel electrophoresis (Invitrogen, E-Gel, 1.2%) was used to confirm the results. Purified DNA samples (20 μl solvent) of fresh and decellularized scaffolds were analyzed and documented (ENDURO GDS, Labnet; Omega Fluor, software).

### Cell culture

Mouse embryonic NIH-3T3 fibroblasts (passages 33–53) stably expressing GFP-actin (NIH3T3-GFP-actin) were cultured in DMEM growth medium (GM) consisted of Dulbecco’s Modified Eagle Media-high glucose with glutamine (DMEM-HG), supplemented with 10% fetal bovine serum, 1% l-Glutamin, 0.1% Penicillin–Streptomycin Solution (50 units/ml penicillin, and 50 μg/ml streptomycin), 1% Sodium Pyruvate solution, 1% non-essential amino acids, (all from Biological Industries, IL), in the 37 °C, 5% CO_2_ incubator. The GM was changed twice a week. Seeding was induced when a confluence of 80% was reached.

### Seaweed cellulose scaffold sterilization

Decellularized cellulose from *Ulva* sp. and *Cladophora* sp. species were sterilized and pretreated prior to experiments. Single seaweed cellulose samples were placed in individual wells and soaked in 70% Ethanol (1 ml/well) overnight at RT°C, in a tissue culture flow hood. Samples were then washed in ultrapure water (UPW) (UltraPure DNsese/RNase-Free, Biolab-Chemicals), three times, then soaked overnight (2 ml/well) at RT°C, in a tissue culture flow hood. Next, the samples were treated in PBS (Dulbecco’s Phosphate Buffered Saline (−) Calcium (−), Magnesium, Biological Industries) (1 ml/well) and incubated overnight (37 °C, %5CO_2_). Finally, the samples were treated in GM consisting of DMEM-HG (1 ml/well), and incubated overnight (37 °C, %5CO_2_). Successively, the media was discarded, and the samples were dried in a tissue culture flow hood, before recellularization seeding took place.

### Recellularization of seaweed cellulose

Following the decellularization treatment, acellular SC scaffolds were recellularized with NIH3T3-GFP-actin cell culture to evaluate in vitro cell growth over a period of time. For the observational analysis tests, non-uniformed sized sterilized samples of the SC scaffolds (1–2 mm^2^ dimensions area) were placed into a new non-treated 24-well plate (SPL Life Sciences). Single samples were placed in individual wells. Following, 5 µl of cell suspension at concentration of 5, 10, 20 and 40 × 10^3^ cells/µl were seeded onto each scaffold and incubated (37 °C, %5CO_2_) for 3 h to allow for initial cell adhesion onto the scaffolds. Following the initial incubation, 1 ml GM consisting of DMEM-HG, was added into each well and resume incubation. Growth media was changed every other day. Cells were observed on the SC scaffolds for up to 8 weeks before fixation with 4% formaldehyde (PFA, Biological Chemicals) took place. All experiments had three replicates. Positive controlled samples of cell and scaffold without cells, as well as controlled blank samples were observed and analyzed for this study.

### Analysis and characterization

#### Scanning electron microscopy (SEM) analysis

Decellularized and recellularized SC scaffolds were evaluated and analyzed using scanning electron microscopy (SEM) (JCM-6000, JEOL, Life Sciences, Tel Aviv University). Samples before and after cell seeding were visualized and recorded at × 50, 130, 400, 650, 1000, 1700, 4000 and × 7000 magnification. SEM images of the SC scaffolds, recellularized with NIH3T3 cell culture, were taken four weeks post seeding. Pore size, cell wall width, fiber diameter and cell culture morphology were observed and determined using image analysis software ImageJ (ImageJ v. 1.51, NIH). To determine the *Ulva* sp. pore size, 50 regions of the interest (ROI) were identified in a given SEM image of the decellularized sample, 10 ROI were identified to determine the *Ulva* sp. cell wall thickness, 55 ROI were identified to determine *Cladophora* sp. fiber width and 50 ROI were identified to determine *Cladophora* sp. microfibrils overlay width. Moreover, 40 ROI were identified to determine the average cell size on the *Ulva* sp. scaffold and 70 ROI were identified to determine the average cell size on the *Cladophora* sp. scaffold. The mean dimensions and standard deviation are reported.

#### Confocal analysis

Real-time monitoring of the cell culture took place with fluorescence confocal microscopy (ZEISS LSM 510META). Images at week 5–6 recorded the NIH3T3-GFP-actin filaments (Argon gas laser 488 nm) and detected the scaffold reflection signal (633 nm). Cell growth was observed and analyzed with Zen (ZEISS microscopy) microscopy image processing and Imaris (Oxford Instruments). Additional time-lapse imaging (20x) of cell growth on the *Ulva* sp. cellulose scaffolds at Day 32 and on the *Cladophora* sp. cellulose scaffolds Day 40 took place.

### Biocompatibility evaluation

Following ISO standard 10993-parts 5 and 12, direct and indirect extract methods were used to evaluate in vitro cytotoxicity of macroalgae cellulose-based scaffolds *Ulva* sp. and *Cladophora* sp. Direct contact test allows for cell seeding directly onto the SC scaffolds, while indirect contact test method was carried out with cell culture incubated in media extracts from the SC scaffolds. Samples were evaluated and analyzed for this study. The mean cell metabolic activity and standard deviation are reported for each test.

#### alamarBlue assay

alamarBlue assay (BioRad, Enco, IL) was used to study and monitor the 3T3 mammalian cell culture viability in the presence of SC based scaffolds over time, following the manufacturer’s protocol. alamarBlue (AB) detects the level of oxidation–reduction (REDOX) during respiration, by detecting the alteration of resazurin, fluorescent blue indicator dye that undergoes colorimetric change into resorufin, fluorescent pink, in response to cellular metabolic reduction. Thus, the increase in AB fluorescence signal over time is used as an indicator of fibroblasts metabolic activity, which is correlated indirectly to cell viability, expressed in cell proliferation and overall cell growth. Following the AB assay, cells were incubated in a 96 well plate with 10% v/v AB solution (200 µl p/well). Successively, duplicates of 100 µl solution samples were carefully distributed into a new 96 well plate. The percentage reduction of the AB dye was measured using a spectrophotometer microplate reader (Thermo Scientific, Multiscan Go) at 570 nm and 600 nm absorbance wavelength. Results were recorded using SkanIt Software.

#### Scaffold cytotoxicity: indirect contact test with alamarBlue assay

Cytotoxic evaluation of *Ulva* sp. and *Cladophora* sp. SC scaffolds took place following ISO 10993-12, Biocompatibility Testing of Medical Devices, sample preparation for the “most severe” surface-area to volume exposure (6 cm^2^ per 1 ml surface area, < 0.5 mm thickness). Accordingly, sterilized *Ulva* sp. and *Cladophora* sp. SC scaffolds were fabricated (weight: 0.3845 g and 0.3493 g, thickness: 0.2–0.35 mm and 0.25–0.30 mm, respectively) and incubated (37 °C, %5C0_2_) in DMEM GM for 24 h on a shaker (20 rpm). Concurrently, fibroblasts at cell density of 10 × 10^3^ cells p/well, were seeded and incubated for 12 h in a 96 well plate. The following day, media was extracted from each scaffold and filtered with 0.22 µm filters, to avoid remaining scaffold fragments. Cells were then incubated with 100% and 30% concentrations of media extracts for 24 h. Subsequently, absorbance measurements were taken after 4 h of incubation with 10% AB solution. Cytotoxicity evaluation was performed before and after the treatment with the media extracts, at the initial state (t = 0) and after 24, 48 and 72 h of incubation (t = 24, 48, 72), for both test groups. Additional control groups, including cells cultured with regular media, blank media and 10% AB solution in media, as well as cytotoxic positive control of 70% Methanol in media (30 min incubation prior to evaluation), were observed and analyzed for this study. The difference in percentage reduction of AB absorption between treated and control samples for each of the SC samples, at each incubation period were calculated and analyzed using the AB percentage difference equation (BioRad):

1 $$Percentage\,difference = \frac{{\left( {O2 \times A1} \right) - \left( {O1 \times A2} \right)}}{{\left( {O2 \times P1} \right) - \left( {O1 - P2} \right)}} \times 100,$$

where O1 and O2 represent the molar extinction coefficient (E) of the oxidized alamarBlue at 570 and 600 nm, respectively. A1 and A2 represent the absorbance of the test wells at 570 and 600 nm, respectively, P1 and P2 represent the absorbance of positive growth control well (cells and alamarBlue solution but no test agent—0% extract) at 570 nm and 600 nm, respectively.

#### Cell viability: direct contact test with alamarBlue assay

*Ulva* sp. and *Cladophora* sp. cellulose scaffolds were cut into uniformed circles (Ø = 2 mm) with a hole puncher device, sterilized and placed into a 96 well plate, a single scaffold disc per well. Since we are unfamiliar with the cell growth on seaweed cellulose scaffolds, we used different cell densities in order to calibrate and optimize cell proliferation. Thus, following the recellularization method, each scaffold was seeded at an initial cell density of 5, 10, 20 and 40 × 10^3^ cells/µl (n = 3). Additionally, control groups, including scaffolds without cell culture for each SC sample, blank media and 10% AB solution (media and alamarBlue but no cells), were observed and analyzed for this study. Following a 24 h incubation (37 °C, 5%CO_2_), AB assay was used to evaluate the cell culture viability in the presence of macroalgae cellulose scaffolds for a period of 6 weeks. It is worth noting that this method does not assure 100% accuracy detecting only the viability of cells on the SC scaffold alone. Thus, in order to reduce the chance of cell growth on the bottom of the well-plates, the seeded SC samples were transferred to a non-treated 12 well plate for continuous growth. Absorbance was measured after 24 h of incubation (37 °C, 5%CO_2_), with 10% AB solution. Continuous monitoring of the AB signal percentage reduction was performed at established time points (t = 1, 2, 4, 8, 11, 15, 19, 22, 25, 29, 32, 36, and 40 days). The difference in percentage reduction of AB absorption between treated and control samples at each cell density and incubation period, were calculated and analyzed using the AB absorbance percentage reduction equation (BioRad):

2 $$Percentage\, reduction = \frac{{\left( {O2 \times A1} \right) - \left( {O1 \times A2} \right)}}{{ \left( {R1 \times N2} \right) - \left( {R2 - N1} \right)}} \times 100,$$

where O1 and O2 represent the molar extinction coefficient (E) of the oxidized alamarBlue at 570 and 600 nm, respectively. A1 and A2 represent the absorbance of the test well at 570 and 600 nm, respectively, R1 and R2 represent the molar extinction coefficient (E) of reduced alamarBlue (pink) at 570 and 600 nm, respectively, and N1 and N2 represent the absorbance at 570 and 600 nm, respectively, of negative control well.

### Cell growth model

A logistic growth model was fitted to the results from the viability direct contact tests using Eq. (3)^63^:

3 $$N = \frac{{KN_{0} }}{{N_{0} + \left( {K - N_{0} } \right)e^{ - rt} }},$$

where N is the predicted cell viability at time t, K is the cell viability carrying capacity of the scaffold, $N_{0}$ is the cell viability at time $t_{0}$ (all represented by percentage reduction of alamarBlue), *r* is the cell proliferation rate and t (days) is the time since $t_{0}$.

Parameters were determined for each scaffold type and for each initial cell concentration. $K$ was determined as the maximum measured percentage reduction. $t_{0}$ was chosen as the time from which consistent growth was measured and $N_{0}$ was determined as the percentage reduction at time $t_{0}$. r was determined by minimizing the RMSRE, calculated by Eq. (4), using the Microsoft Excel Office 365 solver:

4 $$RMSRE = \sqrt {\frac{{\mathop \sum \nolimits_{i = 1}^{n} \left( {\frac{{N_{PV} - N_{m} }}{{N_{PV} }}} \right)^{2} }}{n},}$$

where $N_{PV}$ is the modeled cell viability at time t, $N_{m}$ is the mean of measured cell viability at time t and n is the number of measurement points.

### Statistical analysis

All experiments were carried out with at least three replicates. Values are presented as the mean ± standard deviation (SD), paired with a two-sample T-test coupled with Fischer’s Combined Probability test. Correlations between morphological parameters were evaluated using Spearman's correlation tests. A value of p < 0.05 was considered statistically significant.

## Supplementary Information

Supplementary Information 1.

Supplementary Video 1.

Supplementary Video 2.

Supplementary Video 3.

Supplementary Video 4.
