# Supplementary material for: Seaweed cellulose scaffolds derived from green macroalgae for tissue engineering
Source: Sci Rep. 2021 Jun 4;11:11843. doi: 10.1038/s41598-021-90903-2 (PMC8178384; doi:10.1038/s41598-021-90903-2)
Supplement: Supplementary file 1 — Supplementary Information 1. [file 41598_2021_90903_MOESM1_ESM.docx]

Supplementary Information:

**Seaweed Cellulose Scaffolds Derived from Green Macroalgae for Tissue Engineering**

Nurit Bar-Shai^1*^, Orna Sharabani-Yosef^2*^, Meiron Zollmann^1^, Ayelet Lesman^3,4,^ *^, Alexander Golberg^1,^^.

^1^ Porter School of Environment and Earth Sciences, Tel Aviv University, Tel Aviv, Israel

^2^ School of Biomedical Engineering, Tel Aviv University, Tel Aviv, Israel

^3^ School of Mechanical Engineering, Tel Aviv University, Tel Aviv, Israel

^4^ The Center for the Physics and Chemistry of Living Systems

*Corresponding authors: nuritbarshai@mail.tau.ac.il, [ornashy@tauex.tau.ac.il](mailto:ornashy@tauex.tau.ac.il), [[ayeletlesman@tauex.tau.ac.il](mailto:ayeletlesman@tauex.tau.ac.il)](mailto:ayeletlesman@gmail.com)

^ Equal senior author contribution

**This PDF file includes:**

Materials and Methods Captions for Movies S1 to 4

**Other Supplementary Materials for this manuscript includes the following:**
Supplementary Movies S1 to 4

Supplementary Figure S1

**Supplementary Movie 1.**

Confocal microscopy time-lapse sequence of cell growth on *Ulva sp*. seaweed cellulose scaffold, day 32. (3.2MB, scaffold and NIH3T3-GFP-actin cells). Scale bar= 50 µm

**Supplementary Movie 2.**

Confocal microscopy time-lapse sequence of cell growth on *Ulva sp.* seaweed cellulose scaffold, day 32. (3.2MB, only NIH3T3-GFP-actin cells). Scale bar= 50 µm.

**Supplementary Movie 3.**

Confocal microscopy time-lapse sequence of cell growth on *Cladophora sp.* seaweed cellulose scaffold, day 42. (1.5MB, scaffold and NIH3T3-GFP-actin cells) . Scale bar= 80 µm

**Supplementary Movie 4.**

Confocal microscopy time-lapse sequence of cell growth on *Cladophora sp.* seaweed cellulose scaffold, day 42 (807KB, only NIH3T3-GFP-actin cells). Scale bar= 80 µm


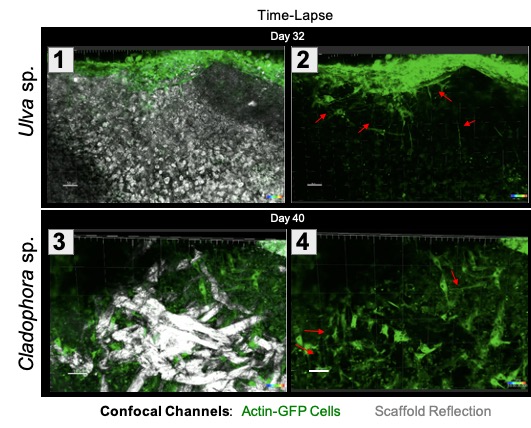


**Supplementary Figure S1-4. Cells growth on seaweed cellulose scaffolds:** Fluorescence confocal microscopy time-lapse imaging (20x), of live fibroblasts (20x10^3^ cells mL^-1.^), labeled with actin-GFP (green), overlay the macroalgae cellulose scaffolds, detected in reflection mode. reveal cell growth and attachments onto the (**S1,2**) *Ulva* sp. porous matrix, (Day 32) and (**S3,4**) *Cladophora* sp. fibrous matrix, (Day 40). Extended slender cell protrusions observed on both scaffolds, indicate that the cells remain alive and function during the entire experiment as they formed connectivity with neighboring cells and the scaffolds’ surface area. *Scale bars: 1,2 = 50µm, 3, 4 = 80µm

**Supplementary Figure S1
DNA quantification analysis:**
SC scaffold were evaluated for their DNA concentrations before and after the decellularization treatment. Fresh and decellularized seaweed samples (Fig. 1A, D, Fig 3F, L) were examined using a plant genomic DNA purification method (n=3 for each sample). DNA concentrations for *Ulva* sp. and *Cladophora* sp. were measured using a NanoDrop spectrophotometer, with 5.53±2.80 ng/µl and 4.18±0.35 ng/µl concentrations, respectively, for the decellularized scaffold samples and 9.59±2.74 ng/µl, and 69.74±16.50 ng/µl concentrations, respectively, for the fresh seaweed samples **(Fig. S1A)*.*** Furthermore, Gel Electrophoresis was used to determine the concentration results for both fresh and decellularized samples **(Fig. S1B).** The results confirm low concentrations for both SC decellularized scaffolds and validated high DNA content for both fresh samples, yet very blurry results for the fresh *Ulva* sp. samples, which validates the low concentration values revealed in the DNA concentration plot.


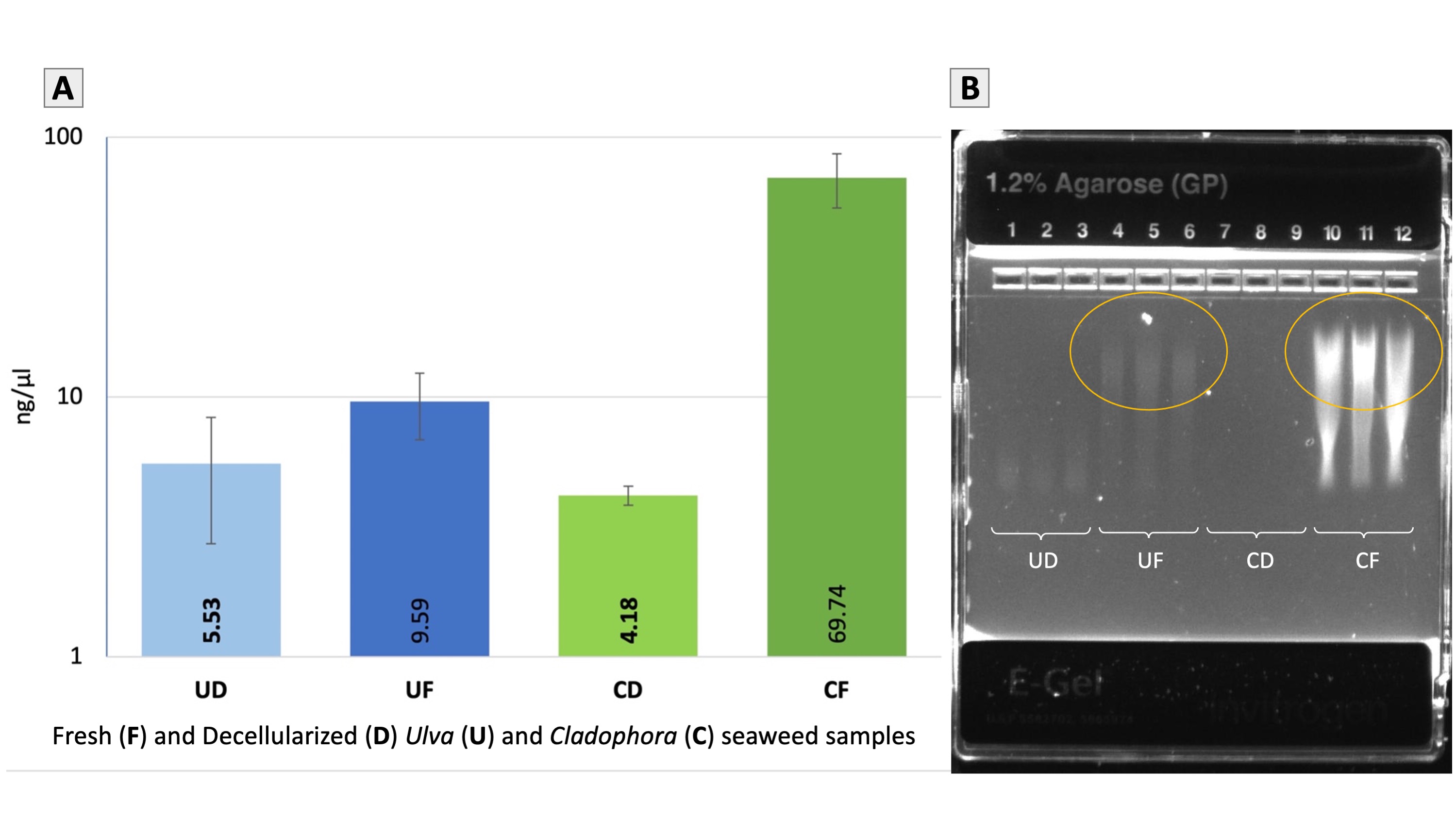
**Supplementary Figure S1. DNA quantification analysis:** SC scaffold were evaluated for their (**A**) Fresh and decellularized seaweed samples (Fig. 1A, D, Fig 3F, L) were examined using a plant genomic DNA purification method (n=3 for each sample). DNA concentrations for *Ulva* sp. and *Cladophora* sp. were measured using a NanoDrop spectrophotometer, with 5.53±2.80 ng/µl and 4.18±0.35 ng/µl concentrations, respectively, for the decellularized scaffold samples and 9.59±2.74 ng/µl, and 69.74±16.50 ng/µl concentrations, respectively, for the fresh seaweed samples***.*** Furthermore, **(B**) Gel Electrophoresis was used to determine the concentration results for both fresh and decellularized samples**.**Yellow circles indicate the high DNA concentrations of the fresh seaweed samples*.* These results confirm low concentrations for both SC decellularized scaffolds and validated high DNA content for both fresh samples, yet very blurry results for the fresh *Ulva* sp. samples, which validates the low concentration values that were revealed in the DNA concentration plot.
